# Lymphatic Absorption of Microbial Plasmalogens in Rats

**DOI:** 10.3389/fcell.2022.836186

**Published:** 2022-03-22

**Authors:** Nana Sato, Aki Kanehama, Akiko Kashiwagi, Miwa Yamada, Megumi Nishimukai

**Affiliations:** ^1^ Faculty of Agriculture, Department of Biological Chemistry and Food Science, Iwate University, Morioka, Japan; ^2^ Faculty of Agriculture and Life Science, Hirosaki University, Hirosaki, Japan; ^3^ Agri-Innovation Center, Iwate University, Morioka, Japan; ^4^ Department of Animal Science, Faculty of Agriculture, Iwate University, Morioka, Japan

**Keywords:** ethanolamine plasmalogen, *Selenomonas ruminantium*, lymphatic absorption, molecular species, microbial plasmalogen

## Abstract

Plasmalogens, functional glycerophospholipids with biological roles in the human body, are associated with various diseases. Although a variety of saturated and/or unsaturated fatty acids in plasmalogens are presumed to have different functions in the human body, there are limited reports validating such functions of plasmalogens. In this study, we focused on the bacterial plasmalogen derived from *Selenomonas ruminantium* subsp. *lactilytica* (NBRC No. 103574) with different main species of hydrocarbon chains at the *sn*-1 position and shorter fatty acids at the *sn*-2 position than animal plasmalogens. Optimum culture conditions of *S. ruminantium* for high-yield production of plasmalogens, such as pH and the concentration of caproic acid, were investigated under anaerobic conditions using a 2-L scale jar fermenter. The obtained plasmalogen mainly consisted of the ethanolamine plasmalogen (PlsEtn). The molar ratios of PlsEtn species obtained from *S. ruminantium*, at *sn*-1/*sn*-2 positions, were p16:1/14:0 (68.4%), p16:1/16:1 (29.2%), p16:1/16:0 (0.7%), p16:1/15:0 (0.3%), and p17:1/14:0 (0.3%). Subsequently, duodenal infusion of the emulsion carrying the lipid extracted from *S. ruminantium* was carried out in lymph duct-cannulated rats. In the lymphatic plasmalogen of rats, the level of PlsEtns with molar ratios p16:1/14:0 and p16:1/16:1, the main species of plasmalogens from *S. ruminantium*, increased gradually until 3–4 h after lipid injection and then gradually decreased. In addition, the level of PlsEtns with p16:1/20:4 and p16:1/22:6 rapidly increased, peaking at 1–1.5 h and 1.5–2 h after lipid injection, respectively. The increase in the number of PlsEtns with p16:1/20:4 and p16:1/22:6 suggested that 20:4 and 22:6, the main fatty acids at the *sn*-2 position in the rat lymphatic plasmalogen, were preferentially re-esterified at the *sn*-2 position, regardless of the types of hydrocarbon chains at the *sn*-1 position. Thus, we showed that bacterial PlsEtns with “unnatural” structures against rats could be absorbed into the lymph. Our findings provide insights into the association between the chemical structure of plasmalogens and their biological functions in humans.

## Introduction

Glycerophospholipids, the main components of biological membranes, play important roles in maintaining the structure and function of biological membranes, as well as in the overall physiology of the organism. They are classified into three subclasses: diacyl, alkyl, and alkenyl, based on the aliphatic hydrocarbon chain at the *sn*-1 position of the glycerol backbone, *via* ester, ether, and vinyl-ether binding, respectively.

**FIGURE 1:**
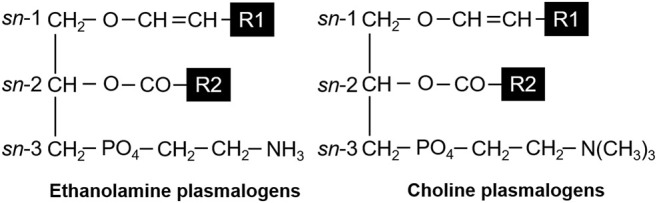
Structures of ethanolamine plasmalogens (PlsEtn) and choline plasmalogens (PlsCho). R1 and R2 denote the carbon chain at the *sn*-1 and *sn*-2 positions, respectively.

Plasmalogens that we focused on the present study are a subclass of glycerophospholipids containing a vinyl-ether bond at the *sn*-1 position and an ester bond at the *sn*-2 position of the glycerol backbone (Figure 1). The *sn*-1 and -2 positions consist of saturated and/or unsaturated fatty acids (FAs) with various carbon chains. It is well known that plasmalogens are present in human and animal tissues (Bradley et al., 2015). In the brain and lungs, the content of ethanolamine plasmalogen (PlsEtn), which has ethanolamine bound to the phosphate group at the *sn*-3 position, is relatively higher than that in other organs (Panganamala et al., 1971; Heymans et al., 1983). In addition, the heart contains choline plasmalogen (PlsCho), which has choline bound to the phosphate group at the *sn*-3 position (Panganamala et al., 1971; Heymans et al., 1983).

Multiple physiological functions of plasmalogens have been presumed: 1) structural components of the cell membrane (Lee, 1998; Nagan and Zoeller, 2001), 2) maintaining cell membrane dynamics (Koivuniemi, 2017), 3) storage compounds of arachidonic acid, a precursor of functional lipid mediators (Garg and Haerdi, 1993), and 4) antioxidants. The antioxidant effect was confirmed by both *in vivo* and *in vitro* radical scavenger examination of reactive oxygen species, and was found to be a result of the vinyl-ether double bond of plasmalogens (Hahnel et al., 1999a; Maeba and Ueta, 2003; Wu et al., 2019). It is not certain whether this antioxidative ability of plasmalogens is the main cause or not, but many previous studies have reported that the composition of plasmalogens is strongly related to the etiopathogenesis of many kinds of diseases (Ginsberg et al., 1995; Hahnel et al., 1999b; Maeba et al., 2007; Khan et al., 2008; Nishimukai et al., 2014a; Nishimukai et al., 2014b). For instance, the presence of plasmalogens in the serum reportedly prevented arteriosclerosis, and a deficiency of plasmalogens was involved in nerve degeneration in Alzheimer’s disease (Goodenowe et al., 2007; Su et al., 2019). Thus, understanding of the physiological function of plasmalogens can provide clarity on disease pathogenesis and/or its prevention.

Plasmalogens are contained in the cell membrane of not only animals but also some strictly anaerobic bacteria such as *Selenomonas ruminantium*, *Ruminococcus albus*, *Ruminococcus flavefaciens*, *Bacteroides succinogenes*, *Borrelia* sp., and *Clostridium butyricum*, which are classified as rumen bacteria (Kamio et al., 1969). In particular, *S. ruminantium* has been well studied as a typical bacterium which contains plasmalogens in the cell membrane (Kamio et al., 1970). Kanegasaki and Takahashi demonstrated that plasmalogens are derived from FA present in the medium containing glucose and ^14^C-labeled FAs used for cultivating *S. ruminantium* and the phospholipid (PL) fraction obtained from ethanol–ether extraction of the cultivated cells (Kanegasaki and Takahashi, 1968). Furthermore, it was reported that the type of energy source in the culture medium affects the plasmalogen content in *S. ruminantium* (Kamio et al., 1969; Kim et al., 1970; Takatsuka and Kamio, 2004)*.* The plasmalogen content of *S. ruminantium* cultivated in a medium containing lactate was approximately three times higher than that of the cells cultivated in a medium containing glucose. Although microbial cells mainly contain PlsEtns, a difference in the chemical structure is found between microbial and animal plasmalogens. Microbial plasmalogens have FAs with a shorter carbon chain length at the *sn*-1 and -2 positions compared to animal plasmalogens (Watanabe et al., 1982). Thus, microorganisms are a good source of plasmalogens which consist of FAs different from that of animal plasmalogens.

In this study, our final goal is to elucidate the physiological structure–function correlation of plasmalogens in humans by comparing the function of plasmalogens having “unnatural” chemical structures against mammals. We investigated optimum large-scale culture conditions, such as the concentration of FAs and pH, to obtain high productivity of microbial plasmalogens derived from *S. ruminantium*. However, plasmalogens are unstable compounds and easily broken under acidic conditions, such as in the stomach, because of its vinyl-ether bond at the *sn*-1 position (Figure 1). We assumed to utilize capsules containing plasmalogens to absorb intact plasmalogens into the small intestine in our future experiments. It is important to perform not only general gavage experiments but also lymphatic absorption experiments. Thus, the lymphatic absorption of microbial plasmalogens from the small intestine was studied using lymph duct-cannulated rats as a first step toward our goal.

## Materials and Methods

### Strain and Culture Conditions

*S. ruminantium* subsp. *lactilytica* (NBRC No. 103574) (Kaneko et al., 2015) was obtained from the National Institute of Technology and Evaluation. The seed culture was carried out in 100-ml sealed vials containing 100 ml of the 988TYG medium (pH 7.0) at 37°C for 24 h under anaerobic conditions. Eighty milliliters of the culture was inoculated into a 2-L jar fermenter (M-1000B, EYELA, Tokyo, Japan) containing 2.1 L of the 988TYG medium and then incubated at 37°C with N_2_ gas flowing. The 988TYG medium contained 0.2% trypticase, 0.2% yeast extract, 0.5% glucose, 0.02% caproic acid sodium salt, 0.3% Na_2_CO_3_, 0.09% KH_2_PO_4_, 0.09% NaCl, 0.09% (NH_4_)_2_SO_4_, 0.001% MnCl_2_·4H_2_O, 0.001% CoCl_2_·6H_2_O, 0.001% CaCl_2_·2H_2_O, and 0.0001% resazurin. After air in the 988TYG medium was partially replaced with N_2_, the medium was autoclaved, and then, 0.2 ml (for 100 ml medium) or 4.2 ml (for 2.1 L medium) of filter-sterilized L-cysteine hydrochloride solution (250 mg/ml) was aseptically added to the medium using syringes.

### Extraction of Lipids From *S. ruminantium* and Porcine Brain

After cultivation, the cells were harvested by centrifugation (6,400 g × 15 min, 4°C) and washed thrice with distilled water. The microbial cells or porcine brain were lyophilized using an FD-1000 vacuum freeze dryer (EYELA, Japan) at −80°C for 2 days. Lipids were extracted with chloroform/methanol/1% KCl solution (1.1: 1.1: 1, by volume), followed by extraction with chloroform twice. All extract solutions were collected, evaporated to dryness, and dissolved in methanol. This fraction was analyzed by ultra-performance liquid chromatography–electrospray ionization tandem mass spectrometry (UPLC/ESI-MS/MS) and subjected to further experiments.

### Preparation of Emulsion

The extracted lipids (25 g/L) from *S*. *ruminantium* and porcine brain in a 1 ml emulsified solution contained 1.83 and 2.77 µmol PlsEtn (estimated average molecular weight, 638 and 768), respectively. The FA composition and molecular species composition of plasmalogens in the PlsEtn are shown in Table 2. These test lipid preparations were emulsified with sodium taurocholate (10 g/L) and triolein (75 g/L) using a sonicator just prior to use.

### Animals

Male Wistar/ST rats (Japan SLC Inc., Hamamatsu, Japan), aged 9 weeks, were fed a standard diet (AIN 93G formula) for a 3-day acclimation period.

After overnight fasting, a vinyl catheter and a silicone catheter were implanted in the thoracic lymph duct and the duodenum, respectively, as described previously (Nishimukai et al., 2011). After the collection of lymph for 30 min (initial lymph) on the day after implanting the indwelling catheter, the rats were administered 1 ml of an emulsified test solution containing 25 mg test lipids for 1 min. The glucose-NaCl isotonic solution without test lipids was infused continuously at 1.8 ml/h through the duodenal tube after the implants of the catheter until the end of the experiment except during an administration of the emulsified test solution.

The lymph was collected from the thoracic duct lymph over time, that is, at 0.5 h intervals during the first 2 h and at 1-h intervals during the next 7 h, following the administration of the test solution. The collected lymph was frozen immediately and kept at –80°C until subsequent analyses.

This study was approved by the Iwate University Animal Committee (approval number; A201450), and animals were maintained in accordance with the Iwate University guideline for the care and use of laboratory animals.

### Analyses

Total lipids in the lymphatic fluid were extracted by the Bligh & Dyer method (Bligh and Dyer, 1959). All plasmalogens in the lipid extracts were analyzed by UPLC/ESI-MS/MS, as previously described (Nishimukai et al., 2011; Nishimukai et al., 2014b). Briefly, liquid chromatography (LC) separation was performed using a Dionex UltiMate 3000 system (Thermo Fisher Scientific, Waltham, MA, United States) with a BEH C8 column (1.7 µm, 100 mm × 2.1 mm I.D.; Waters Corp., Milford, MA, United States) at 60°C and a flow rate of 0.45 ml/min. The mobile phase A consisted of water containing 5 mM ammonium formate, and the mobile phase B consisted of acetonitrile. Mass spectrometry (MS) analysis was performed using a TSQ Quantum Access MAX instrument (Thermo Fisher Scientific) equipped with an ESI probe in the positive ion mode. We checked many molecular species in a preliminary experiment and selected those in order of the quantity contained in the sample. PlsEtn was quantified according to the principles described (Zemski Berry and Murphy, 2004). In brief, PlsEtn was identified by fragments derived from the *sn*-1 position (p16:0, p16:1, p17:0, p17:1, and p18:1). “p” indicates the carbon chain of *sn*-1 in plasmalogens with a vinyl-ether linkage. PlsCho was identified by three fragments and was quantified by phosphocholine as a fragment ion at m/z 184 after the separation of each PlsCho molecule by UPLC. In addition, the presence of plasmalogens was confirmed by the disappearance of the peak after treatment with acid. The parent and fragment ions of the plasmalogen molecular species measured in this study are shown in Table 1. The collision energy for PlsCho and PlsEtn was 32 and 18 eV, respectively.

**TABLE 1:** The parent and CID (collision induced dissociation) fragment ions of the measured plasmalogen molecular species.

PlsEtn				PlsCho			
*sn*-1	*sn*-2	[M + H]^+^	CID fragment ion	*sn*-1	*sn*-2	[M + H]^+^	CID fragment ion
p16:0	14:0	648.49	364	p16:0	14:0	690.54	184
	15:0	662.50	364		15:0	704.55	184
	15:1	660.49	364		15:1	702.54	184
	16:0	676.52	364		16:0	718.57	184
	16:1	674.50	364		16:1	716.55	184
	17:0	690.54	364		17:0	732.58	184
	17:1	688.52	364		17:1	730.57	184
	18:0	704.55	364		18:0	746.60	184
	18:1	702.54	364		18:1	744.58	184
	18:2	700.52	364		18:2	742.57	184
	20:4	724.52	364		20:4	766.57	184
	20:5	722.51	364		20:5	764.55	184
	22:4	752.55	364		22:4	794.60	184
	22:5	750.54	364		22:5	792.58	184
	22:6	748.52	364		22:6	790.57	184
p16:1	14:0	646.47	362	p16:1	14:0	688.52	184
	15:0	660.48	362		15:0	702.53	184
	15:1	658.47	362		15:1	700.52	184
	16:0	674.50	362		16:0	716.55	184
	16:1	672.48	362		16:1	714.53	184
	17:0	688.52	362		17:0	730.56	184
	17:1	686.50	362		17:1	728.55	184
	18:0	702.53	362		18:0	744.58	184
	18:1	700.52	362		18:1	742.56	184
	18:2	698.51	362		18:2	740.55	184
	20:4	722.51	362		20:4	764.55	184
	20:5	720.49	362		20:5	762.54	184
	22:4	750.54	362		22:4	792.58	184
	22:5	748.52	362		22:5	790.57	184
	22:6	746.51	362		22:6	788.55	184
p17:0	14:0	662.51	378	p17:0	14:0	704.56	184
	15:0	676.52	378		15:0	718.57	184
	15:1	674.51	378		15:1	716.56	184
	16:0	690.54	378		16:0	732.59	184
	16:1	688.52	378		16:1	730.57	184
	17:0	704.56	378		17:0	746.60	184
	17:1	702.54	378		17:1	744.59	184
	18:0	718.57	378		18:0	760.62	184
	18:1	716.56	378		18:1	758.60	184
	18:2	714.54	378		18:2	756.58	184
	20:4	738.54	378		20:4	780.58	184
	20:5	736.52	378		20:5	778.57	184
	22:4	766.57	378		22:4	808.61	184
	22:5	764.55	378		22:5	806.60	184
	22:6	762.54	378		22:6	804.58	184
p17:1	14:0	660.49	376	p17:1	14:0	702.54	184
	15:0	674.50	376		15:0	716.55	184
	15:1	672.49	376		15:1	714.54	184
	16:0	688.52	376		16:0	730.57	184
	16:1	686.50	376		16:1	728.55	184
	17:0	702.54	376		17:0	744.58	184
	17:1	700.52	376		17:1	742.57	184
	18:0	716.55	376		18:0	758.60	184
	18:1	714.54	376		18:1	756.58	184
	18:2	712.52	376		18:2	754.57	184
	20:4	736.52	376		20:4	778.57	184
	20:5	734.51	376		20:5	776.55	184
	22:4	764.55	376		22:4	806.60	184
	22:5	762.54	376		22:5	804.58	184
	22:6	760.52	376		22:6	802.57	184
p18:0	14:0	676.52	392	p18:0	14:0	718.57	184
	15:0	690.53	392		15:0	732.58	184
	15:1	688.52	392		15:1	730.57	184
	16:0	704.55	392		16:0	746.60	184
	16:1	702.53	392		16:1	744.58	184
	17:0	718.57	392		17:0	760.61	184
	17:1	716.55	392		17:1	758.60	184
	18:0	732.58	392		18:0	774.63	184
	18:1	730.57	392		18:1	772.61	184
	18:2	728.55	392		18:2	770.60	184
	20:4	752.55	392		20:4	794.60	184
	20:5	750.54	392		20:5	792.58	184
	22:4	780.58	392		22:4	822.63	184
	22:5	778.57	392		22:5	820.62	184
	22:6	776.55	392		22:6	818.60	184
p18:1	14:0	674.51	390	p18:1	14:0	716.56	184
	15:0	688.52	390		15:0	730.57	184
	15:1	686.51	390		15:1	728.56	184
	16:0	702.54	390		16:0	744.59	184
	16:1	700.52	390		16:1	742.57	184
	17:0	716.56	390		17:0	758.60	184
	17:1	714.54	390		17:1	756.59	184
	18:0	730.57	390		18:0	772.62	184
	18:1	728.56	390		18:1	770.60	184
	18:2	726.54	390		18:2	768.58	184
	20:4	750.54	390		20:4	792.58	184
	20:5	748.52	390		20:5	790.57	184
	22:4	778.57	390		22:4	820.61	184
	22:5	776.55	390		22:5	818.60	184
	22:6	774.54	390		22:6	816.58	184

Synthetic p18:0–18:1 and p18:0–20:4 of PlsCho and PlsEtn, (Avanti Polar Lipids, Alabaster, AL, United States) were used to generate a standard curve to quantify individual species of ether glycerophospholipids. Total PlsEtn and PlsCho were taken as the sum of all molecular species in each class measured by UPLC-MS/MS. The lipid composition in the lipid extracts from bacteria and porcine brain was measured by thin layer chromatography on chromarods with flame ionization detection (FID) using an Iatroscan MK-6 instrument (Mitsubishi Chemical Yatron, Tokyo, Japan) (Watanabe et al., 2019).

### Calculation and Statistical Analysis

Data are expressed as mean ± SEM (standard error of the mean). The results were analyzed by one-way analysis of variance followed by Dunnett multiple comparisons. Data analysis was performed by the add-in software Statcel 3 (OMS, Tokyo, Japan). Differences with *p* < 0.05 were taken to be statistically significant.

## Results

### Effects of Caproic Acid Concentration and pH on Biosynthesis of PlsEtn in *S. ruminantium*

**FIGURE 2:**
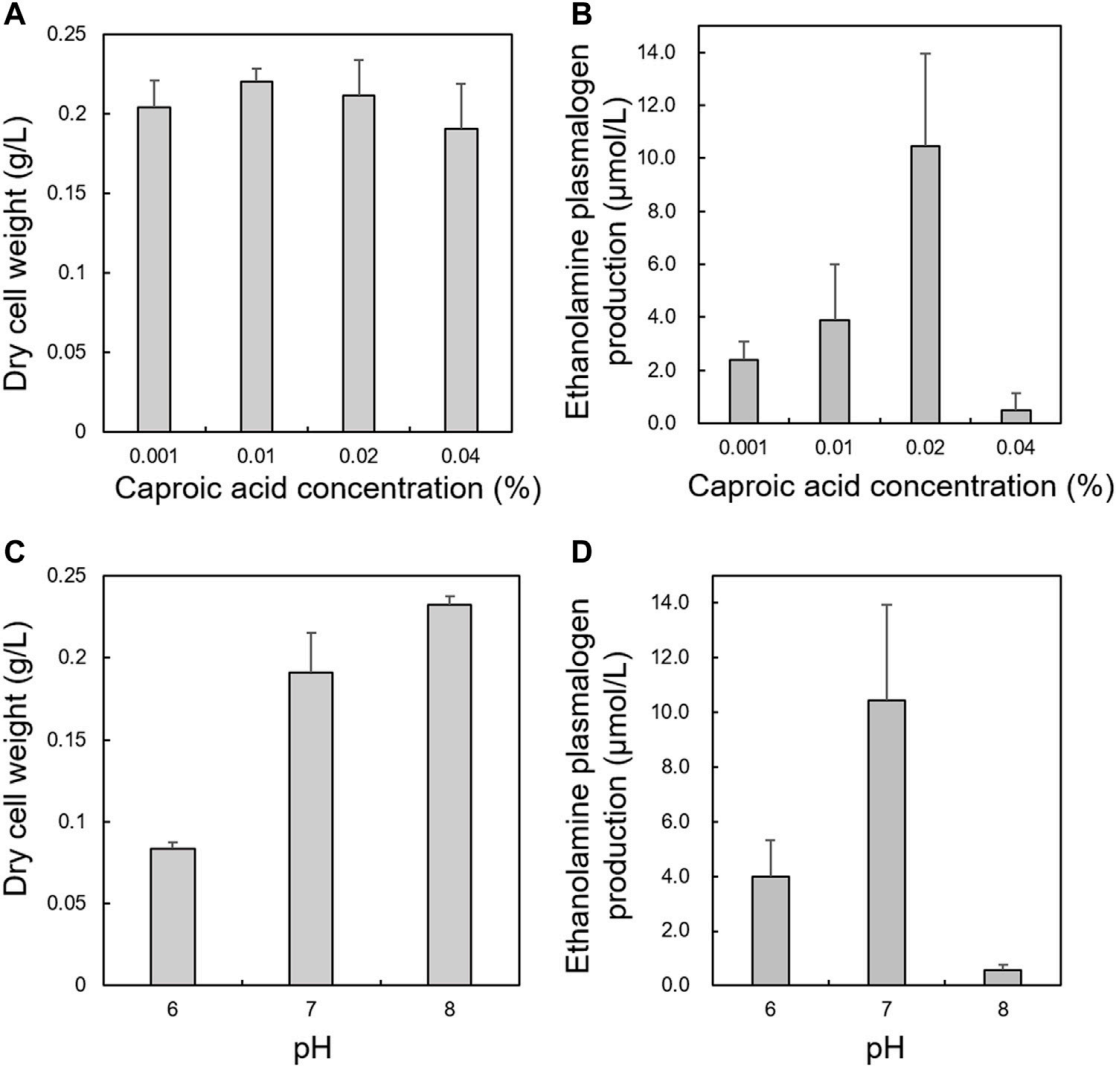
Effects of caproic acid concentration and pH on growth and PlsEtn production in *S. ruminantium*. Effect of caproic acid at different concentrations on **(A)** growth of *S. ruminantium* and **(B)** PlsEtn production of *S. ruminantium* in the glucose medium at 37°C and pH 7.0 for 24 h. Effect of pH on **(C)** the growth of *S. ruminantium* and **(D)** PlsEtn production of *S. ruminantium* in the glucose medium containing 0.02% caproic acid at 37°C for 24 h. Dry cell weight was measured after freeze drying. PlsEtn production was measured by LC-MS/MS analysis of lipids extracted from dry cells. The data represent mean ± SD (*n* = 3).

First, we optimized culture conditions such as the concentration of caproic acid and pH in a glucose medium for 2.1-L scale anaerobic cultivation of *S. ruminantium.* The effects from 0.001 to 0.04% caproic acid in the glucose medium (component is similar to the 988TYG medium except to concentration of caproic acid sodium salt) were examined at 37 °C and pH 7.0 for 24 h. Although there was no effect on the cell growth at different caproic acid concentrations (0.001–0.04%) (Figure 2A), PlsEtn production per 1 L culture broth was the maximum in the glucose medium containing 0.02% caproic acid (Figure 2B). Subsequently, we investigated the effect of pH on growth and PlsEtn production when *S. ruminantium* was cultured in the glucose medium containing 0.02% caproic acid at 37°C for 24 h. Growth increased along with pH from 6 to 8 (Figure 2C), but PlsEtn production was the highest at pH 7 (Figure 2D). The maximum PlsEtn production was 10.5 ± 3.5 μmol/L of culture broth, and the molar ratios of PlsEtn species were p16:1/14:0 (68.4%), p16:1/16:1 (29.2%), p16:1/15:0 (0.3%), p16:1/15:1 (0.1%), p16:1/16:0 (0.7%), and p17:1/14:0 (0.3%) (Table 2). Furthermore, phosphatidyl ethanolamine including PlsEtn accounted for 73% of the lipid extract from *S. ruminantium* (Table 3).

**TABLE 2:** The composition of fatty acids in the lipid extracts and molecular species composition in ethanolamine plasmalogen of lipid extracts from *Selenomonas ruminantium* and porcine brain.

Origin			
			Fatty acids (%)
*S. ruminantium*	14:0		26.71
	15:0		0.51
	16:0		11.38
	16:1		42.56
	17:1		2.53
	18:0		1.27
	18:1		7.18
	18:2		1.78
	Others		6.09
			100
	*sn*-1 Alkenyl	*sn*-2 Acyl	Ethanolamine plasmalogen (%)
	p16:1	14:0	68.4
	p16:1	15:0	0.3
	p16:1	15:1	0.1
	p16:1	16:0	0.7
	p16:1	16:1	29.2
	p17:1	14:0	0.3
	Others		1.0
			100.0
			Fatty acids (%)
Porcine brain	16:0		31.22
	16:1		2.55
	17:1		3.38
	18:0		21.77
	18:1		22.88
	18:1		5.07
	20:4		5.44
	22:5		1.30
	22:6		2.73
	Others		3.66
			100
	*sn*-1 Alkenyl	*sn*-2 Acyl	Ethanolamine plasmalogen (%)
	p18:1	18:1	21.4
	p18:1	20:4	13.7
	p18:0	22:6	12
	Others		52.9
			100

**TABLE 3:** The composition (%) of lipids in the lipid extracts from *S. ruminantium* and porcine brain.

	Amount of lipid (%) in lipid extracts	
	*S. ruminantium*	Porcine brain
PE	73	40.4
PC + SM	—	23.3
ChoE + TG	3.59	4.87
Chol	3.66	22.3
Others	19.75	9.13
	100	100

PE, phosphatidyl ethanolamine including PlsEtn; PC + SM, phosphatidyl choline + sphingomyelin; ChoE + TG, cholesterolester + triglyceride; Chol, cholesterol.

### Lymphatic Absorption of PlsEtn Derived From *S. ruminantium* and Porcine Brain in Rats

To examine the characteristic of absorption of plasmalogens derived from *S. ruminantium*, the lymphatic output of plasmalogens in rats administered with bacterial plasmalogens from *S. ruminantium* or porcine brain was analyzed using the UPLC-MS/MS method and compared. In the lipid extract from porcine brain, the molar ratios of PlsEtn species were p18:1/18:1 (21.4%), p18:1/20:4 (13.7%), and p18:0/22:6 (12%) (Table 2). Phosphatidyl ethanolamine including PlsEtn accounted for 40.4% of the lipid extract (Table 3). In addition, phosphatidyl choline and sphingomyelin including PlsCho accounted for 23.3% of the lipid extract.

**FIGURE 3:**
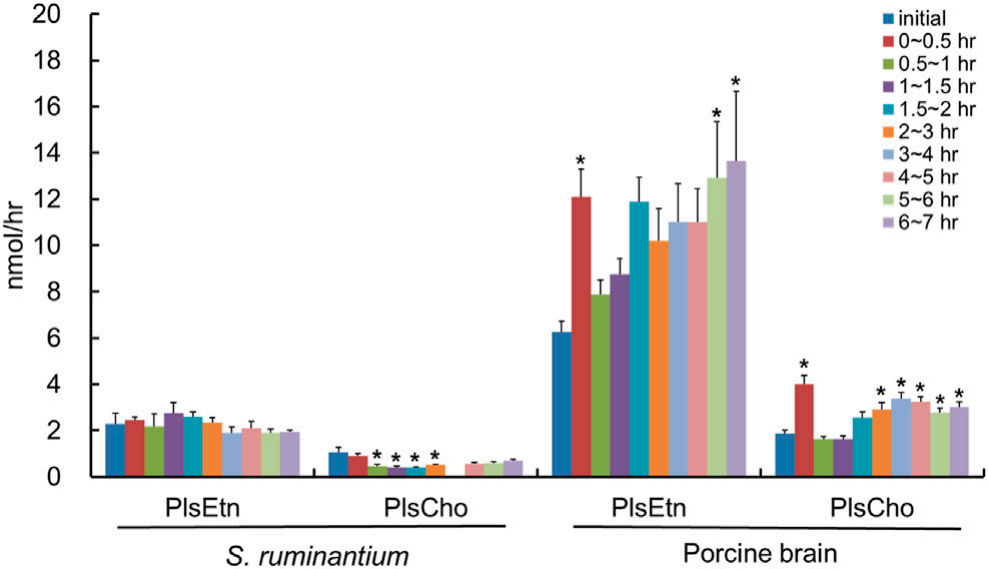
Total amounts of PlsEtn and PlsCho in the lymph of thoracic lymph-cannulated rats for 7 h after duodenal administration of the emulsion containing lipids extracted from *S. ruminantium* or porcine brain. Lipids extracted from *S. ruminantium* and porcine brain were adjusted to the same amount in total lipids when the duodenal administration was conducted. Data are represented as mean ± SEM (*n* = 5–6). Asterisk indicates significant difference with respect to the values at time 0 (*p* < 0.05).

**FIGURE 4:**
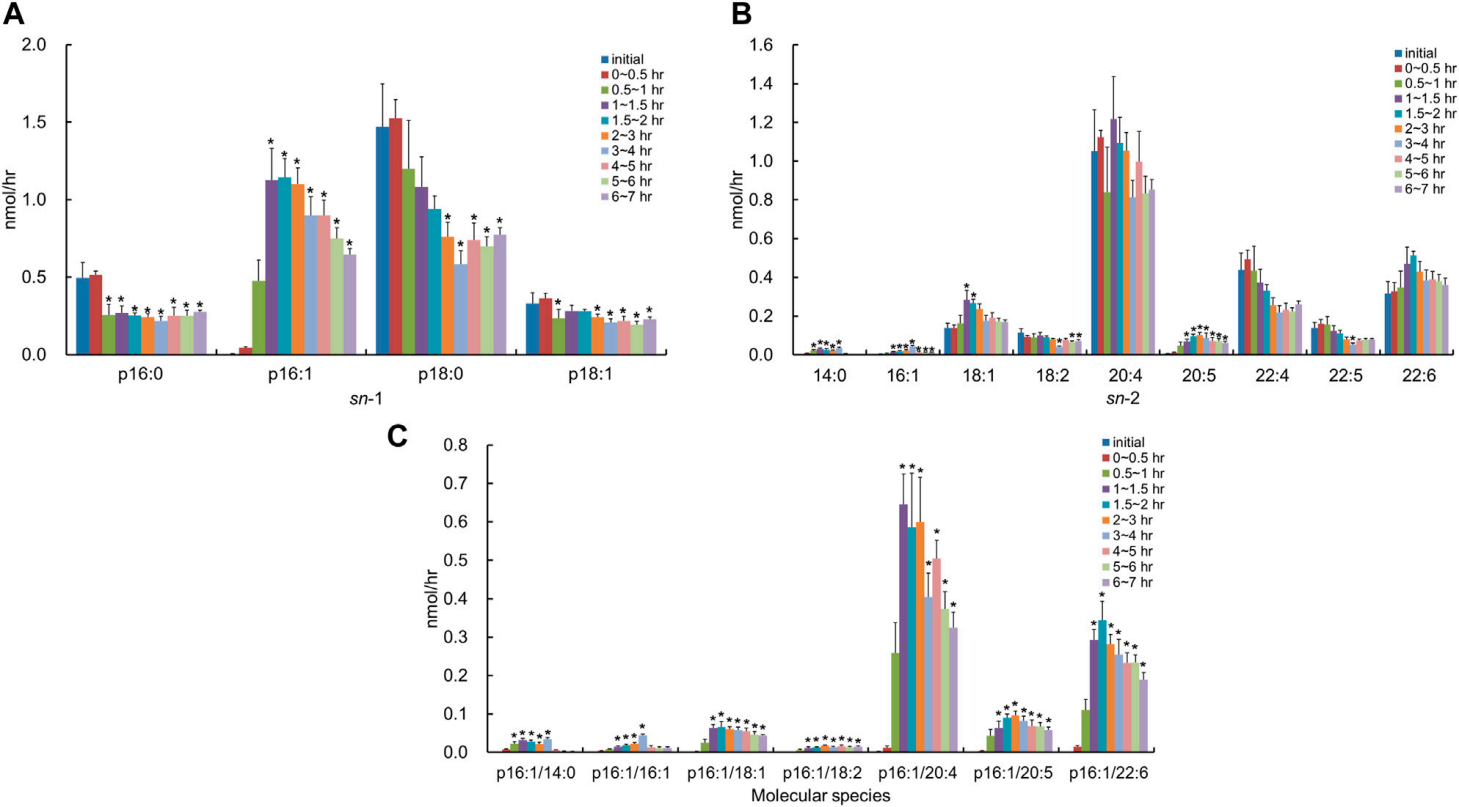
Change in molecular species due to the classification in **(A)** the hydrocarbon chain at the *sn*-1 position and **(B)** FA at the *sn*-2 position and **(C)** change in molecular species with p16:1 at the *sn*-1 position, in lymphatic PlsEtn of thoracic lymph-cannulated rats for 7 h after duodenal administration of the emulsion containing the lipid extracted from *S. ruminantium*. Data are represented as mean ± SEM (*n* = 5–6). Asterisk indicates significant difference with respect to the values at time 0 (*p* < 0.05).

After the injection of the lipid derived from *S. ruminantium* containing the PlsEtn, the total PlsEtn output in the lymph was not significantly increased compared to that prior to the administration of the lipid emulsion containing plasmalogens (initial) (Figure 3). However, the level of PlsEtn containing p16:1 at the *sn*-1 position was increased, reaching a peak value at 1–4 h (Figure 4A). The molecular species of PlsEtn with p16:1 at *sn*-1 detected using the UPLC-MS/MS method were p16:1/14:0, p16:1/16:1, p16:1/18:1, p16:1/18:2, p16:1/20:4, p16:1/20:5, and p16:1/22:6 (Figure 4C). The amounts of PlsEtns with p16:1/14:0 and p16:1/16:1 peaked at 3–4 h and nearly returned to the initial level at 4–5 h. Furthermore, the levels of PlsEtn with p16:1/20:4, p16:1/22:6, p16:1/18:1, and p16:1/20:5 peaked at 1–3 h after administration and remained higher than the initial level thereon; the levels of PlsEtns, especially with p16:1/20:4 and p16:1/22:6, rapidly increased. However, the amounts of PlsEtn containing p16:0, p18:0, and p18:1 at the *sn*-1 position decreased over time and remained at levels lower than that of the initial level up to 7 h (Figure 4A). The molar ratios of the hydrocarbon chain at the *sn*-1 position of PlsEtn in the lymph are as follows: 21.4%, p16:0; 0.2%, p16:1; 64.3%, p18:0; and 14.1%, p18:1 for the initial lymph, and 9.9%, p16:0; 43.3%, p16:1; 35.9%, p18:0; and 10.9%, p18:1 at 1–1.5 h after the administration of plasmalogens from *S. ruminantium*, which were mostly maintained up to 7 h. With regards to FAs at the *sn*-2 position, the amounts of PlsEtn with 18:2, 22:4, and 22:5 decreased after administration, whereas those with 14:0, 16:1, 18:1, and 20:5 increased (Figure 4B).

**FIGURE 5:**
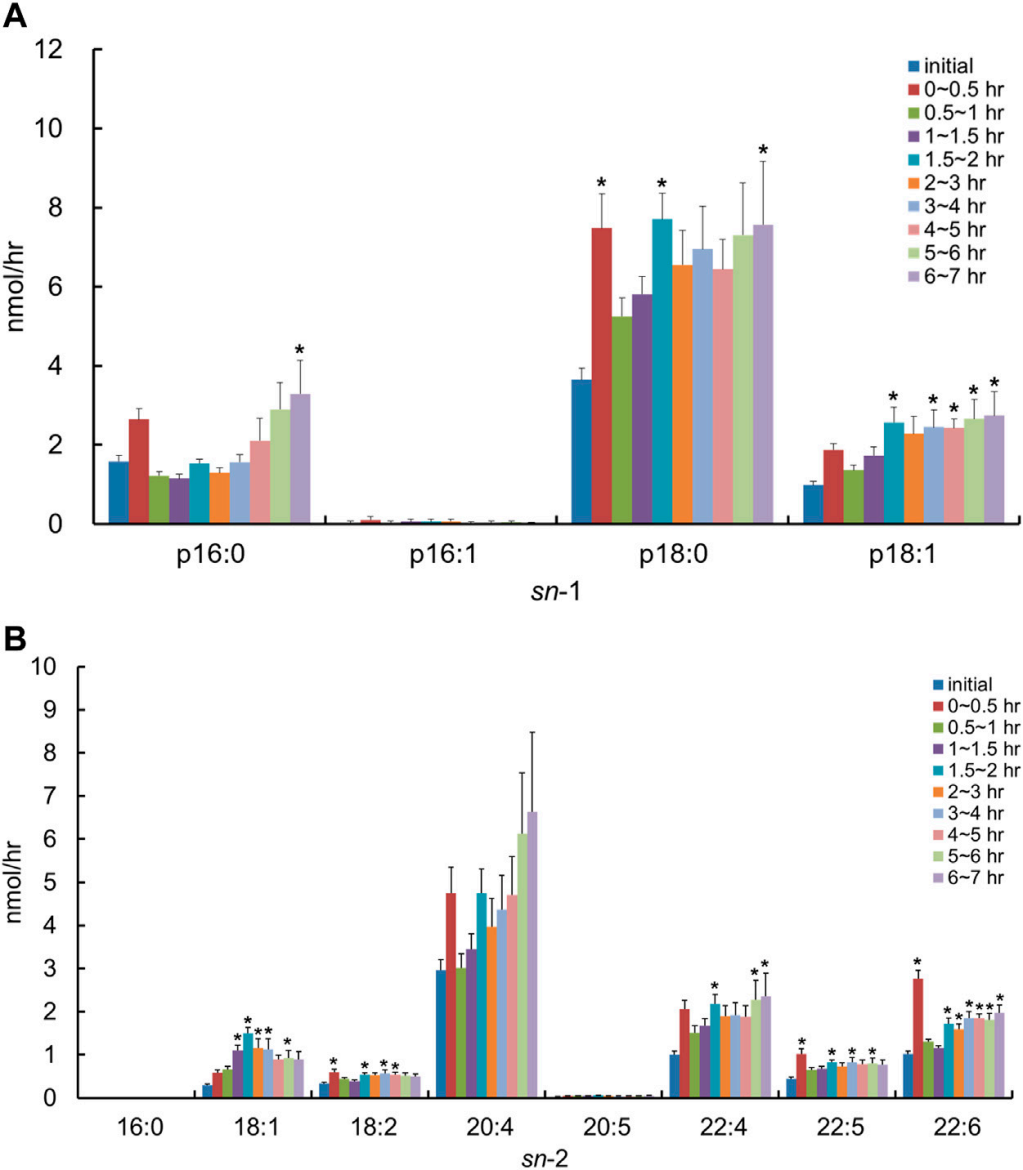
Change in molecular species due to the classification in **(A)** the hydrocarbon chain at the *sn*-1 position and **(B)** FA at the *sn*-2 position in lymphatic PlsEtn of thoracic lymph-cannulated rats for 7 h after duodenal administration of the emulsion containing the lipid extracted from porcine brain. Data are represented as mean ± SEM (*n* = 5–6). Asterisk indicates significant difference with respect to the values at time 0 (*p* < 0.05).

We used the lipid extract from porcine brain, which is known to be rich in PlsEtn, as a comparison control. After the injection of the lipid derived from porcine brain, the molecular species of PlsEtn containing p16:1 at the *sn*-1 position was barely detected in the lymph, and the level of PlsEtn containing p16:0, p18:0, and p18:1 at the *sn*-1 position increased, particularly that of 18:0 (Figure 5A). As for the molar ratios of the hydrocarbon chain at the *sn*-1 position of the PlsEtn, the levels of the PlsEtn with p18:0 and p18:1 at the *sn*-1 position increased after administration and that of p16:0 decreased as follows: 25.2%, p16:0; 0.5%, p16:1; 58.3%, p18:0; and 15.8%, p18:1 for the initial lymph, and 13.1%, p16:0; 0.5%, p16:1; 66.6%, p18:0; and 19.8%, p18:1 at 1–1.5 h. However, the ratios were returning to a level closer to that as the initial lymph as follows: 24.2%, p16:0; 0.2%, p16:1; 55.5%, p18:0; and 20.1%, p18:1 at 6–7 h. Furthermore, with regard to FAs at the *sn*-2 position, the amounts of almost all PlsEtns were time-dependently increased compared to that in the initial lymph; in particular, the level of the molecular species with 20:4 at the *sn*-2 position increased (Figure 5B). As a result, the total PlsEtn output in the lymph was increased time-dependently by the injection of the porcine brain lipid (*p* = 0.050) (Figure 3).

The total lymphatic outputs of PlsEtn for 7 h after the administration of plasmalogens derived from *S. ruminantium* and porcine brain were 6.2 ± 0.5 nmol and 33.0 ± 6.5 nmol, respectively. The absorption percentage of total PlsEtn from *S. ruminantium* into the lymph was approximately one-fourth compared to that derived from the porcine brain (0.33 ± 0.03% for *S. ruminantium* and 1.19 ± 0.23% for porcine brain).

### Lymphatic Absorption of PlsCho Derived From *S. ruminantium* and Porcine Brain in Rats

**FIGURE 6:**
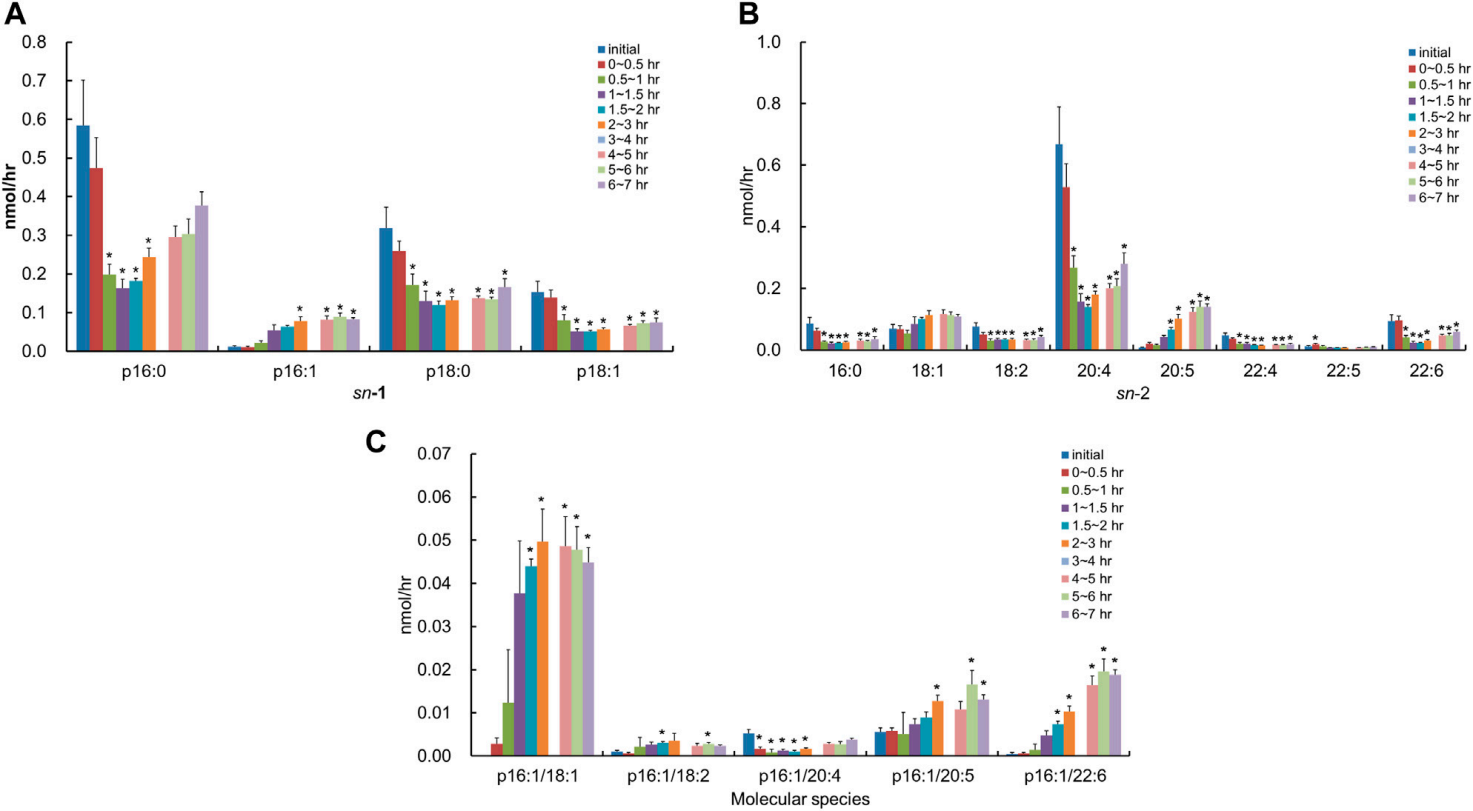
Change in molecular species due to the classification in **(A)** the hydrocarbon chain at the *sn*-1 position and **(B)** FA at the *sn*-2 position and **(C)** change in molecular species with p16:1 at the *sn*-1 position in the lymphatic PlsCho of thoracic lymph-cannulated rats for 7 h after duodenal administration of the emulsion containing the lipid extracted from *S. ruminantium*. Data are represented as mean ± SEM (*n* = 5–6). Asterisk indicates significant difference with respect to the values at time 0 (*p* < 0.05). The data of 3–4 h were missing in order to mistake analyzing.

After the injection of the lipid derived from *S. ruminantium* containing PlsEtn, the lymphatic output of total PlsCho was decreased and then gradually recovered by half of the initial value at 6–7 h (Figure 3). The level of PlsCho containing p16:0, p18:0, and p18:1 at the *sn*-1 position reached its lowest at 1–2 h after administration and then gradually increased (Figure 6A). On the other hand, the level of PlsCho containing p16:1 at the *sn*-1 position, particularly that of p16:1/18:1, reached the peak value at 2–3 h after administration and remained high until 7 h (Figure 6A); however, p16:1/14:0 and p16:1/16:1 were not detected (Figure 6C). The molar ratios of the hydrocarbon chain at the *sn*-1 position of PlsCho in the initial lymph are as follows: 54.7%, p16:0; 1.1%, p16:1; 29.8%, p18:0; and 14.4%, p18:1, whereas those in the lymph 1–1.5 h after plasmalogen (from *S. ruminantium*) administration are as follows: 41.0%, p16:0; 13.5%, p16:1; 32.6%, p18:0; and 12.9%, p18:1. Moreover, only the p16:1 ratio was maintained until after 6–7 h. With regard to FAs at the *sn*-2 position, PlsCho with 16:0, 18:2, 20:4, 22:4, 22:5, and 22:6 was decreased after administration of porcine plasmalogens, whereas those with 18:1 and 20:5 was increased (Figure 6B).

**FIGURE 7:**
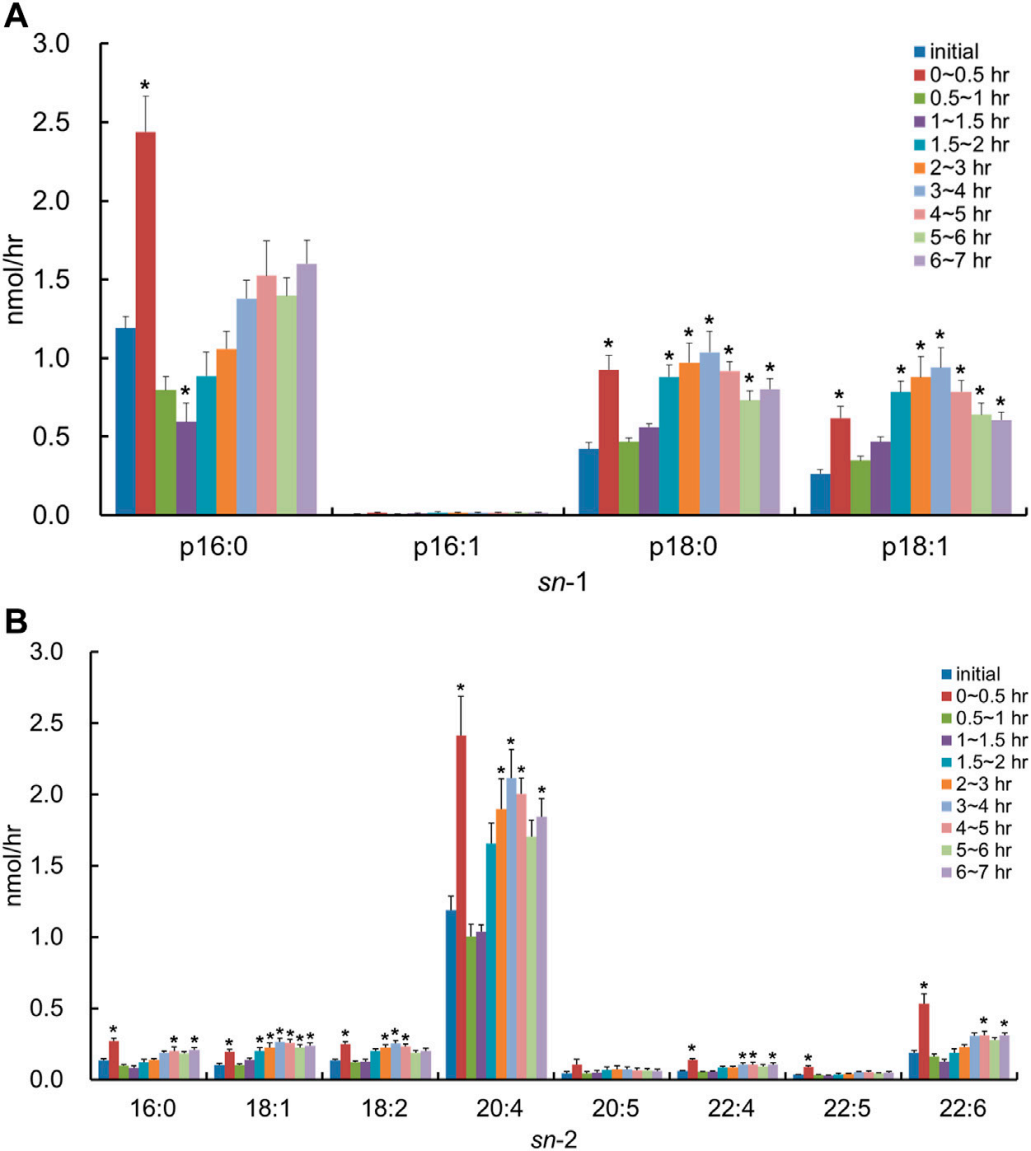
Change in molecular species due to the classification in **(A)** the hydrocarbon chain at the *sn*-1 position and **(B)** FA at the *sn*-2 position in the lymphatic PlsCho of thoracic lymph-cannulated rats for 7 h after duodenal administration of the emulsion containing the lipid extracted from porcine brain. Data are represented as mean ± SEM (*n* = 5–6). Asterisk indicates significant difference with respect to the values at time 0 (*p* < 0.05).

In contrast, after the injection of the lipid derived from porcine brain containing PlsEtn, the molecular species of PlsCho containing p16:1 at the *sn*-1 position was barely detected in the lymph, and the level of PlsCho with p16:0, p18:0, and p18:1 increased to the same extent time-dependently (Figure 7A). With regard to FAs at the *sn*-2 position, the lymphatic output of almost all PlsCho, and especially that of the molecular species with 20:4 at the *sn*-2 position, was time-dependently increased compared to the initial lymph, regardless of the type of FA binding at the *sn*-2 position in PlsCho (Figure 7B). There was almost no change in the composition of both *sn*-1 and *sn*-2 in PlsCho after the injection of the lipid extract from porcine brain.

There were no significant differences in the amount of lymph flow among rats administered with each plasmalogen emulsion (data not shown).

## Discussion

The results of plasmalogen production by *S. ruminantium* in the present study show differences when compared to results of other previous experiments. A previous study showed that glucose media containing volatile FAs or lactate media containing biotin were appropriate for the growth of *S. ruminantium* (Kanegasaki and Takahashi, 1967). However, in the present study, *S. ruminantium* grew well in the glucose medium and not so much in the lactate medium (data not shown). In addition, the production of serine plasmalogens (PlsSer) was not detected in our study, although it is reported that PlsEtn and PlsSer are the main plasmalogens produced in *S. ruminantium* (Watanabe et al., 1982). These differences could be due to differences in culture conditions such as the culture volume and anaerobic level.

**FIGURE 8:**
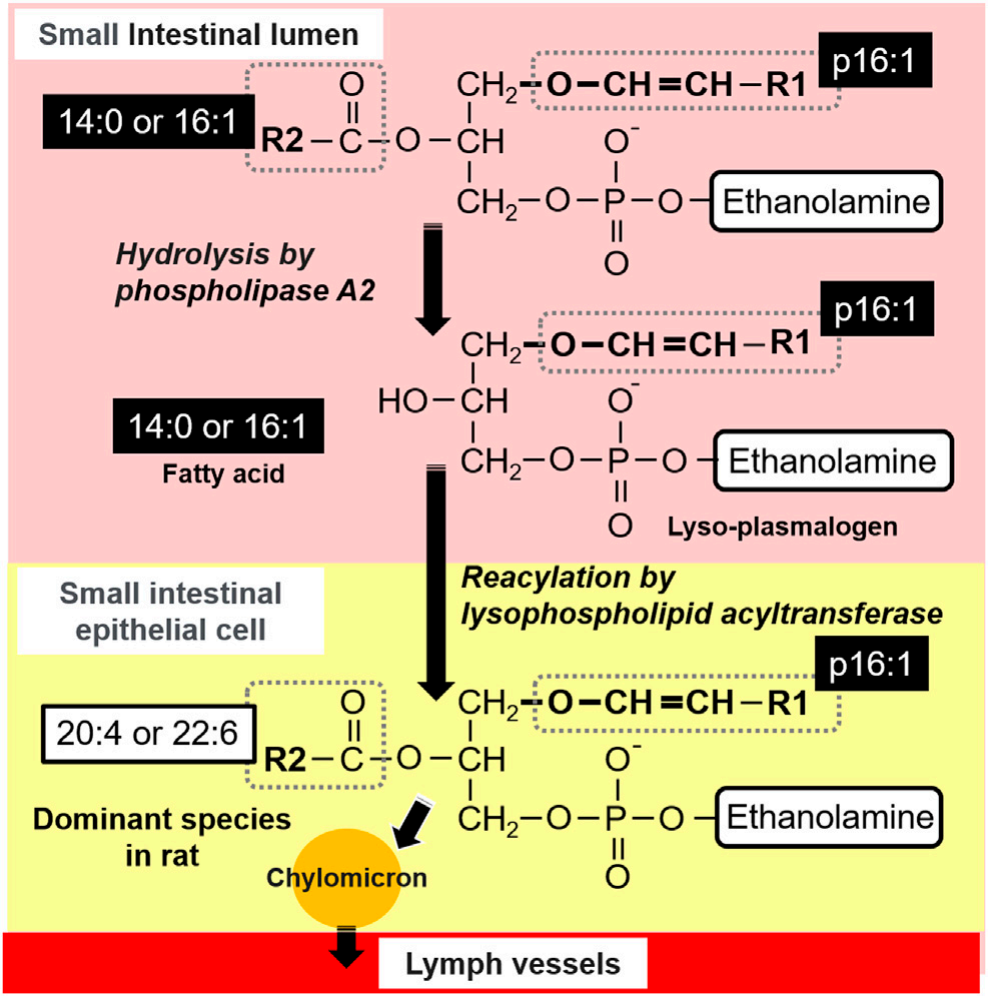
Predicted lymphatic adsorption of plasmalogens from *S. ruminantium* in rats.

In general, in the small intestine, most of the dietary PLs are hydrolyzed at the *sn*-2 position by pancreatic phospholipase A2 (pPLA2) and then absorbed by the intestinal mucosal cells as free FAs and lyso-phospholipids (Lyso-PLs) in the small intestinal lumen (Figure 8). Lyso-PLs and some free-FAs are re-esterified to PLs by acyltransferase in the small intestinal epithelial cells and released into the lymph flow incorporated in chylomicrons. However, it has been assumed that some intestinal PLs are absorbed passively and without hydrolyzation (Scow et al., 1967; Zierenberg and Grundy, 1982). The most common PL present in food is phosphatidylcholine, whereas other PLs, such as phosphatidylethanolamine, phosphatidylserine, and phosphatidylinositol, are present in much smaller amounts (Murru et al., 2013). Therefore, although there are more reports about phosphatidylcholine compared to other PLs, it is reported that phosphatidylethanolamine goes through similar stages of digestion and absorption as phosphatidylcholine, despite their differences in the efficiency of digestion and uptake (Ikeda et al., 1987; Nishimukai et al., 2011). Based on this knowledge, it is considered that plasmalogens are absorbed in the small intestine through a process similar to that of the absorption of other PLs.

After administration of the lipid from *S. ruminantium* to rats, the level of PlsEtn containing the p16:1 hydrocarbon chain at the *sn*-1 position increased in the lymph (Figure 4A). The PlsEtn with p16:1 at *sn*-1 is characteristic of PlsEtn from *S. ruminantium* and is barely detected from the initial lymph fluid of rats under normal diet. In contrast, after the injection of the lipid from porcine brain, the level of PlsEtn with p18:0 and p18:1 at the *sn*-1 position, which is the main hydrocarbon chain at the *sn*-1 position of plasmalogens in mammalian tissues such as brain (Nishimukai et al., 2011), increased without an increase in the level of PlsEtn with the p16:1 hydrocarbon chain. Thus, the compositions of the *sn*-1 position of plasmalogens in the lymph roughly reflected the composition of the administered plasmalogens, which suggests that at least part of microbial plasmalogens administered to rats was absorbed from the small intestine and released into the lymph while maintaining the molecular structure of *sn*-1 (Figure 8).

The level of PlsEtn with p16:1/20:4 and p16:1/22:6 (Figures 4, 8) increased significantly in the lymph compared to that in the initial lymph after administration of plasmalogens from *S. ruminantium*. Lipids from *S. ruminantium* were rich in 14:0 and 16:1 but not in 20:4 and 22:6 (Table 2). On the other hand, FAs of the *sn*-2 position in PlsEtn in the lymph were very rich in 20:4 and 22:6 but only slightly in 14:0 and 16:1. Similarly, after the administration of plasmalogens from porcine brain, nearly half of the FAs at the *sn*-2 position in the lymphatic plasmalogens were 20:4 and 22:6 and did not reflect the FA composition of the injected lipid. From these results, we confirmed that 20:4 and 22:6 were preferentially re-esterified at the *sn*-2 position during absorption of PlsEtn in the small intestine, regardless of the types of carbon chain at the *sn*-1 position. Previously, studies conducted by us and others have shown that PlsEtn preferentially selected 20:4 for re-esterification in the intestine using plasmalogens with p16:0, p18:0, and p18:1 at the *sn*-1 position (Nishimukai et al., 2011; Takahashi et al., 2020). However, the present study is the first to show that plasmalogens containing p16:1, which is rarely found at the *sn*-1 position in animal plasmalogens, have similar characteristics to those containing p16:0, p18:0, and p18:1. The level of PlsEtn with 20:5 at the *sn*-2 position (Figure 4B) also increased significantly in the lymph compared to that in the initial lymph after administration of plasmalogens from *S. ruminantium*. This phenomenon is interesting because the abundance of 20:5 in phospholipids is generally at the low level in the phospholipids. However, we have no reasonable reasons until now.

The structural change, which is the replacement of FAs with 20:4 and 22:6, is possibly involved in FA turnover during PL remodeling by reacylation of lyso-PL by lysophospholipid acyltransferases (LPLATs) (Cao et al., 2008; Harayama et al., 2014; Bradley et al., 2015; Hashidate-Yoshida et al., 2015; Eto et al., 2020). Lyso-phosphatidylcholine acyltransferase 3 (LPCAT3) is highly expressed in the small intestine and involved in incorporating 20:4 into PLs (Hashidate-Yoshida et al., 2015). Further, lyso-phosphatidylethanolamine acyltransferase 2 (LPEAT2) preferentially incorporates 22:6 into PLs in mouse brain cells, unfortunately but not the report about LPEAT2 in the small intestine (Cao et al., 2008; Eto et al., 2020). Though not certain, it is possible that these lysophospholipid acyltransferases equally act for the re-esterification of PlsEtn. As mentioned earlier, most of the PlsEtn absorbed in the small intestine was released into the lymph as PlsEtn with polyunsaturated FAs (20:4 and 22:6) at the *sn*-2 position. However, the levels of PlsEtn having p16:1/14:0 and p16:1/16:1 were also increased in the lymph significantly (Figure 4B). The ratio of these species was 4.3% of the total PlsEtn released into the lymph after administration of plasmalogens from *S. ruminantium*. This result may be consistent with the activity of lysophospholipid acyltransferase mentioned previously. It is possible that lysophospholipid acyltransferases in the rat intestine act weakly in incorporating 14:0 and 16:1 into the *sn*-2 position in PlsEtn. Further studies are required to evaluate the structural change that where and how the ingested lipids are re-esterified.

The absorption rate of PlsEtn derived from *S. ruminantium* (0.33 ± 0.03%) into the lymph for 7 h after administration was lower than that of PlsEtn derived from porcine brain (1.19 ± 0.23%). This might be a result of the LPLAT activity for PlsEtn with p16:1 being weaker than that for PlsEtn with p18:0 and p16:0 during re-esterification. Moreover, bacterial lipids consist of relatively short-chain FAs with less than 16 carbons such as 14:0 and 16:1, while pig brain lipids consist of long-chain FAs with more than 18 carbons including 20:4 and 22:6 (Table 2). There are many kinds of PLAs, which hydrolyze FAs at the *sn*-2 position of PLs, with various substrate specificities. Therefore, it is possible that the difference in composition, that is, the number of carbon and double bonds in PlsEtn, has an effect on the activity of phospholipase A2 for PlsEtn. However, the detailed mechanisms are still unclear. Therefore, further study is needed to clarify the relation between these enzymes and the structure of plasmalogens.

The lipid from *S. ruminantium* used in this study was rich in 16:1 (palmitoleic acid), according to the composition analysis using the GC and LC-MS/MS method (Table 2). However, the level of PlsEtn with 16:1 at the *sn*-2 position was not high in the lymph after the injection of the lipid derived from *S. ruminantium* (Figure 4C). In this case, 16:1, which is produced by hydrolysis, is re-esterified to other PLs and triglycerides, and released into the lymph after incorporation into chylomicrons. Consumption of palmitoleic acid prevents hypercholesterolemia, atherosclerosis, fatty liver, and metabolic disorders caused by a high-fat, high-carbohydrate diet (Nunes and Rafacho, 2017; Huang et al., 2020; Souza et al., 2020). Although 16:1 incorporated in the *sn*-2 position of PlsEtn was detected in traces, 16:1 incorporated in other lipids is possibly beneficial to the human body, suggesting new usage of bacterial lipids.

After administration of PlsEtn from *S. ruminantium*, PlsCho with the p16:1 at the *sn*-1 position increased significantly in the lymph, even though PlsCho was absent in the lipid from *S. ruminantium* (Figure 6). This result shows that ingested microbial PlsEtn was converted to PlsCho in the small intestinal absorptive cells. PlsCho is known to be formed from PlsEtn by enzymes such as phospholipase C and choline phosphotransferase (Strum et al., 1992; Strum and Daniel, 1993). Previous reports have also indicated the conversion of PlsEtn to PlsCho in small intestinal cells in rats (Nishimukai et al., 2011; Takahashi et al., 2020). Furthermore, we have confirmed such conversion in Caco-2 cells (data not shown). Furthermore, the administration of lipids from *S. ruminantium*, containing PlsEtn, time-dependently increased the level of PlsCho containing p16:1 at the *sn*-1 position, temporarily decreasing the levels of PlsEtn containing p16:0, p18:0, and p18:1 at the *sn*-1 position. Furthermore, the peak lymphatic release of PlsCho with p16:1 at the *sn*-1 position was delayed by about 1 h compared with that of PlsEtn with p16:1 at the *sn*-1 position. This is because, immediately after administration, the re-synthesis of PlsEtn is promoted preferentially according to the amounts of lyso-PlsEtn and FA, following which, the conversion of PlsEtn to PlsCho proceeds. Moreover, unexpectedly, we found that the level of PlsCho with p16:1/18:1 was significantly increased in the lymph after the administration of the lipid from *S. ruminantium* (Figure 6). Previous reports have suggested a strong correlation between blood concentrations of PlsCho having an 18:1 FA bound at the *sn*-2 position and atherosclerosis-related factors, as well as a protective function against atherosclerosis (Nishimukai et al., 2014a; Nishimukai et al., 2014b); the increase in the level of p16:1/18:1 PlsCho in the body after administration of microbial plasmalogens may have some positive effect on atherosclerosis.

In this study, we focused on bacteria as the resource of plasmalogens. However, bacteria such as *S. ruminantium* are not appropriate to food resource, because *S. ruminantium* is classified into gram-negative bacteria containing endotoxin in the outer membrane of their cell walls. We assumed to utilize plasmalogens extracted from bacteria for dietary supplement in future work. Also, both general gavage experiments as well as lymphatic absorption experiments are required to understand the structure–function correlation of plasmalogens. Therefore, in this study, the lymphatic absorption of microbial plasmalogens in the small intestine was investigated using lymph duct-cannulated rats as a first step toward this goal. In the future, owing to the instability of plasmalogens under acidic conditions, such as in the stomach, we will develop how to facilitate delivery of intact plasmalogens into the small intestine as utilization of capsules containing plasmalogens.

## Conclusion

In this study, we focused on *S. ruminantium* as a new source of PlsEtn and investigated the absorption mechanism of bacterial PlsEtns with an “unnatural” structure in lymph-cannulated rats. As a result, despite having different chemical structures, it was suggested that a part of bacteria PlsEtn shows the structural change as similar to animal PlsEtn during intestinal absorption, that is, it undergoes 20:4 and 22:6 re-esterification and base conversion (PlsEtn into PlsCho). This finding is expected to not only lead the effective utilization of bacterial resources as food but also clarify the relationship between the chemical structure of plasmalogens and their biological functions in humans.

## Data Availability

The raw data supporting the conclusion of this article will be made available by the authors, without undue reservation.
